# EhVps35, a retromer component, is involved in the recycling of the EhADH and Gal/GalNac virulent proteins of *Entamoeba histolytica*

**DOI:** 10.3389/fpara.2024.1356601

**Published:** 2024-03-26

**Authors:** Joselin Díaz-Valdez, Rosario Javier-Reyna, Sarita Montaño, Daniel Talamás-Lara, Esther Orozco

**Affiliations:** ^1^ Departamento de Infectómica y Patogénesis Molecular, Centro de Investigación y de Estudios Avanzados del Instituto Politécnico Nacional [CINVESTAV-Instituto Politécnico Nacional (IPN)], Mexico City, Mexico; ^2^ Laboratorio de Bioinformática y Simulación Molecular, Facultad de Ciencias Químico-Bilógicas, Universidad Autónoma de Sinaloa, Sinaloa, Mexico; ^3^ Unidad de Microscopía Electrónica, Laboratorios Nacionales de Servicios Experimentales (LaNSE), Centro de Investigación y de Estudios Avanzados del Instituto Politécnico Nacional [CINVESTAV-Instituto Politécnico Nacional (IPN)], Mexico City, Mexico

**Keywords:** retromer, EhVps35, *Entamoeba histolytica*, EhADH (ALIX family), Gal/GalNac lectin, actin cytoskeleton, phagocytosis, recycling

## Abstract

The retromer is a highly conserved eukaryotic complex formed by the cargo selective complex (CSC) and the sorting nexin (SNX) dimer subcomplexes. Its function is protein recycling and recovery from the endosomes to conduct the target molecules to the trans-Golgi network or the plasma membrane. The protozoan responsible for human amoebiasis, *Entamoeba histolytica*, exhibits an active membrane movement and voracious phagocytosis, events in which the retromer may be fully involved. In this work, we studied the structure of EhVps35 the central member of the CSC retromeric subcomplex as it binds EhVps26 and EhVps29, the other two CSC members, allowing the position of the retromer in the membranes. We also studied the EhVps35 role in the recycling of virulence proteins, particularly those involved in phagocytosis. Confocal microscopy assays revealed that EhVps35 is located in the plasmatic and endosomal membranes and in the phagocytic cups and channels. In addition, it follows the target cell from the moment it is in contact with the trophozoites. Molecular docking analyses, immunoprecipitation assays, and microscopy studies revealed that EhVps35 interacts with the EhADH, Gal/GalNac lectin, and actin proteins. In addition, experimental evidence indicated that it recycles surface proteins, particularly EhADH and Gal/GalNac proteins, two molecules highly involved in virulence. Knockdown of the *Ehvps35* gene induced a decrease in protein recycling, as well as impairments in the efficiency of adhesion and the rate of phagocytosis. The actin cytoskeleton was deeply affected by the *Ehvps35* gene knockdown. In summary, our results revealed the participation of *EhVps35* in protein recycling and phagocytosis. Furthermore, altogether, our results demonstrated the concert of finely regulated molecules, including EhVps35, EhADH, Gal/GalNac lectin, and actin, in the phagocytosis of *E. histolytica*.

## 1 Introduction

The eukaryotic canonical retromer is a cellular structure strongly involved in the endocytic–selecting–recycling pathway. In general, it is composed of five proteins that form two subcomplexes: the cargo selective complex (CSC) shaped by Vps26, Vps29, and Vps35 proteins and the SNX-BAR dimer (Vps5 and Vps17 in yeast) that tubulates the endosomal membrane. However, currently, novel functions for retromer proteins continue to be discovered (Bonifacino and Hurley, 2008; Tomavo et al., 2013; Sangaré et al., 2016; McNally et al., 2017; Gras et al., 2019; Huang et al., 2022). In humans, for example, the decreased retromer function has been implicated in neurodegenerative processes, causing Parkinson’s and Alzheimer’s disease (Zhang et al., 2018).

The Vps35 protein is considered as the retromer central component because it interacts with the other two CSC retromeric components and with proteins such as Rab7 (Ypt7 in yeast) that stabilizes the structure in the endosomal membrane (Seaman et al., 2009; Liu et al., 2012; Purushothaman et al., 2017). It is known that Vps35 participates in the retrograde transport from the endosome to the plasma membrane or the *trans*-Golgi network (TGN), recognizing the cargo proteins for recycling (Bonifacino and Hurley, 2008; Zhang et al., 2018). Silencing of the *vps35* gene inhibits signaling of the mitogen-activated protein kinase and the growth of melanoma cells (Zhou et al., 2016). Based on these findings, Vps35 has been proposed as a putative target for anticancer drugs (Zhou et al., 2016). Knockout of the *vps35* gene causes ultrastructural and functional alterations in lysosomes, leading to impaired autophagy (Cui et al., 2019). The accumulation of pathogenic proteins due to a dysfunctional Vps35 accounts for other physiological disorders (Wang and Bellen, 2015; Zhang et al., 2018; Cui et al., 2019; Huang et al., 2022).

In protozoa, the retromer complex has been poorly studied, although it is known that it differs from the canonical structure that was first described in yeast and then later in mammals (Koumandou et al., 2011). Future investigations on this cellular complex are necessary to yield additional insights into the mechanisms that could be involved in the virulence processes in protozoa. In *Toxoplasma gondii*, a parasite lacking SNX-BAR proteins, the retromer participates in the formation of rhoptry and microneme secretory organelles, which are necessary for intracellular invasion (Tomavo et al., 2013; Gras et al., 2019). In *Entamoeba histolytica*, the protozoan responsible for human amoebiasis, the retromer is composed of the EhVps26, EhVps29, EhVps35, and EhSNX1 proteins (Nakada-Tsukui et al., 2005; Watanabe et al., 2020). *E. histolytica* trophozoites incessantly remodel their membrane during movement, phagocytosis, tissue invasion, and vesicular trafficking; thus, it is logical to assume that its non-canonical retromer is functional and probably participates in virulence processes.

In *E. histolytica*, the EhVps26, EhVps29, and EhVps35 proteins have been detected in phagosomes (Marion et al., 2005; Nakada-Tsukui et al., 2005; Srivastava et al., 2017; Watanabe et al., 2020). In addition, live and immunofluorescence images showed that EhSNX1 and EhVps26 were recruited to the trogocytic cups and tunnel-like structures (Watanabe et al., 2020), suggesting a role of the retromer in target cell ingestion. However, further studies are necessary to clarify the mechanisms that make possible or are required to elucidate the role that each retromer member plays in the full complex.

The aim of this work was to investigate the structure of EhVps35 and its functional role in the recycling of EhADH (a protein belonging to the ALIX family) and the Gal/GalNac lectin, which are both involved in target cell adhesion and in the phagocytic process. Our *in silico* analysis revealed that EhVps35 is a *bona fide* ortholog of the Vps35 proteins described in other eukaryotic systems. In addition, our experimental results using a trophozoite population with the *Ehvps35* gene knocked down showed that this protein participates in plasma membrane protein recycling. Remarkably, we present here evidence that the EhADH protein and Gal/GalNac lectin are recycled by EhVps35. In addition, we found that this protein has an important role in the actin cytoskeleton structuring, which makes it a fundamental player in phagocytosis.

## 2 Materials and methods

### 2.1 *In silico* analysis of EhVps35

*Homo sapiens* and *Saccharomyces cerevisiae* Vps35 protein sequences (access Q96QK1 and P34110, respectively), retrieved from the UniProt database (https://www.uniprot.org), were used as query to search the *E. histolytica* Vps35 proteins (EhVps35) in the AmoebaDB database (https://amoebadb.org/amoeba/app). The structural domains of the candidates were identified using the SMART genomics server (http://smart.embl-heidelberg.de/) and the KEGG server (https://www.genome.jp/kegg/). The predicted amino acid sequences of the five *E. histolytica* putative EhVps35 proteins (access C4M8Z4, C4M4G0, A0A8U0WP17, C4LWG4, and C4M3E8) were aligned with the orthologous sequences of *H. sapiens* (access Q96QK1), *S. cerevisiae* (access P34110), *Arabidopsis thaliana* (access Q7X659, F4I0P8, and A8R7K9), *Plasmodium falciparum* (access Q8IIQ6), *T. gondii* (access S7USD8), *Trypanosoma brucei* (access Q38C17), and *Giardia lamblia* (access Q6Y0Y2).

### 2.2 Plasmid constructs

The complementary DNA (cDNA) was obtained as described (Galindo et al., 2021). The *Ehvps35_1–1,428 bp_* and *Ehvps35_1–543 bp_* DNA fragments were PCR-amplified using cDNA as template and specific primers.

1. For *Ehvps35_1–1,225 bp_*: sense: 5′-CCGGTACCATGAGTCGTCCACAACGAGA-3′; antisense: 5′-GGGGATCCTTT TATATCATCTGGTTGATCTTG-3′
2. For *Ehvps35_1–543 bp_*: sense: 5′-CCGGTACCATG AGTCGTCCACAACGAG-3′; antisense: 5′-GGGGATCCTGTTGGATGTTG AACCGCTC-3′

Underlined sequences correspond to the enzyme restriction sites added to the primers. Fragments were cloned in *pColdI* to produce recombinant proteins tagged with 6× His and in *pL4440* for the silencing experiments. Thus, we generated the *pColdI/Ehvps35_1–1,225 bp_* and *pL4440/Ehvps35* plasmids, respectively. The quality of the constructs was verified by restriction enzyme analysis and automatic DNA sequencing.

### 2.3 Expression of the His-Eh Vps35 recombinant protein

*Escherichia coli* BL21 (DE3) bacteria were transformed with the *pColdI/Ehvps35_1–1,225 bp_* plasmid to produce the His-tagged EhVps35_1–476 aa_ recombinant protein (rEhVps35). Protein expression was induced by 0.4 mM isopropyl β-d-thiogalactopyranoside (IPTG) in Luria–Bertani (LB) medium for 16 h at 16°C. The identity and integrity of the purified rEhVps35 protein were verified by 10% SDS-PAGE and Western blot assays.

### 2.4 Antibodies

To generate mouse polyclonal antibodies against a specific EhVps35 peptide (N_30_-ESEIMNAALNNKDLSK-C_45_), male Balb/c mice (from an already existing collection in UPEAL-CINVESTAV) were immunized with 80 µg of the peptide resuspended in TiterMax Gold adjuvant (1:1; Sigma-Aldrich, St. Louis, MO, USA). Thereafter, three more immunizations were performed at 15-day intervals, followed by bleeding to generate the α-EhVps35 antibody. Pre-immune serum was obtained before immunizations.

The other primary antibodies used were as follows: mouse monoclonal α-biotin (Santa Cruz, Dallas, TX, USA), hamster α-EhADH (Galindo et al., 2022), mouse α-heavy subunit Hgl of Gal/GalNac lectin (kindly given by Dr. William A. Petri Jr., University of Virginia, Charlottesville, VA, USA), mouse α-Hist (Santa Cruz), mouse monoclonal α-human actin (kindly given by Dr. Manuel Hernandez, CINVESTAV IPN, Mexico), and rat α-EhVps23 (Galindo et al., 2021). As secondary antibodies, the following were used: horseradish peroxidase (HRP)-labeled α-hamster, α-rat, and α-mouse immunoglobulin G (IgG) (Zymed, San Francisco, CA, USA) for the Western blot assays and fluorescein isothiocyanate (FITC), Cy5, or the Pacific Blue α-mouse and Alexa 647 α-hamster IgG (Life Technologies, Carlsbad, CA, USA) for the immunofluorescence assays.

### 2.5 *E. histolytica* cultures

*E. histolytica* trophozoites, strain HM1:IMSS, were axenically grown at 37°C in TYI-S-33 medium (Diamond et al., 1978) and harvested at the logarithmic growth phase. Afterward, the culture flasks were chilled at 4°C and the trophozoites collected by centrifugation. All experiments reported here were performed at least three times in duplicate.

### 2.6 Western blot experiments

The trophozoite lysates (35 µg) were electrophoresed in 10% SDS-PAGE, transferred to nitrocellulose membranes, and probed with either α-EhVps35 (1:500), α-Hist (1:500), α-actin (1:3,000), α-Hgl Gal/GalNac lectin (1:100), α-EhADH (1:500), or α-EhVps23 (1:500) antibodies. The membranes were washed, incubated with the respective HRP-labeled secondary antibodies (1:1,000; Sigma), and revealed using the ECL Prime detection reagent (GE Healthcare, Chicago, IL, USA) according to the manufacturer’s instructions.

### 2.7 Laser confocal microscopy assays

Trophozoites were grown on coverslips, fixed with 4% paraformaldehyde at 37°C for 1 h, permeabilized (or not) with 0.2% Triton X-100, and blocked with 10% fetal bovine serum in phosphate-buffered saline (PBS). The preparations were incubated at 4°C overnight (ON) with the primary antibodies (1:50). For some experiments, α-EhVps35 labeled with a FITC fluorochrome kit (Molecular Probes/Thermo Fisher, Waltham, MA, USA) was also used. The preparations were incubated with the corresponding secondary antibodies for 30 min at 37°C: FITC-labeled α-mouse IgG for α-EhVps35, Cy5-labeled α-mouse IgG for α-biotin, Pacific Blue-labeled α-mouse IgG for α-Hgl Gal/GalNac, and Alexa Fluor 647-labeled α-hamster IgG for α-EhADH. The nuclei were labeled with 4′,6-diamidino-2-phenylindole (DAPI) for 5 min at room temperature (RT), and F-actin was detected by incubation with rhodamine phallodin (Invitrogen, Carlsbad, CA, USA) for 20 min at 37°C. All preparations were preserved using a VECTASHIELD antifade reagent (Vector, Newark, CA, USA). Subsequently, 0.5-µm laser sections were obtained and examined through a Carl Zeiss LMS 700 confocal microscope and then processed with ZEN 2009 Light Edition software (Zeiss, Jena, Germany). To evaluate the co-localization between molecules, Pearson’s coefficients were obtained from at least 30 confocal images using ImageJ 1.45v software and the JACoP plug-in.

### 2.8 Phagocytosis assays

For the phagocytosis assays, the trophozoites were incubated at 37°C for 2, 5, and 30 min with red blood cells (RBCs; 1:25) from an already existing collection. At different times, the trophozoites were prepared for immunofluorescence (García-Rivera et al., 1982) and observed through a laser confocal microscope. Other preparations were stained using the Novikoff technique (Novikoff et al., 1972), and adhered or ingested RBCs were counted through a light microscope (Zeiss Axiolab) in 100 randomly chosen trophozoites. In other experiments (i.e., pulse-chase), the trophozoites were incubated with RBCs for 2 min at 37°C. Afterward, the cell mixtures were washed with TYI/water (2:1) for 5 min at 37°C to remove the adhered and non-ingested RBCs. Subsequently, the cells were incubated at 37°C for different times (3, 13, 28, 58, or 88 min) and processed for Western blot or immunofluorescence assays.

To visualize the acidic structures, the trophozoites were incubated with LysoTracker™ Blue DND-22 (2 μg/ml; Invitrogen) at 37°C for 2 h and were then prepared for the immunofluorescence assays as described above. In other cases, to inhibit *de novo* protein synthesis, the trophozoites were incubated for 30 min at 37°C with cycloheximide (CHX; 100 µg/ml), washed with PBS, and then used for the experiments after verifying that the viability was greater than 98%, which was evaluated using trypan blue exclusion (0.2%) and examined under a light microscope (Bañuelos et al., 2012).

### 2.9 Transmission electron microscopy

The trophozoites in basal condition and those incubated for 5 min with RBCs (1:25) at 37°C were prepared for TEM as described (Talamás-Lara et al., 2021). Briefly, the trophozoites were fixed with 4% paraformaldehyde and 0.5% glutaraldehyde in PBS for 1 h at RT, washed with PBS, and dehydrated with increasing concentrations of ethanol. After infiltration, the samples were embedded in LR White resin (London Resin Co., London, UK) and polymerized under UV at 4°C for 48 h to obtain thin sections (60 nm), which were mounted on Formvar-coated nickel grids followed by ON incubation at 4°C with the α-EhVps35 antibody (1:50). Thereafter, the samples were incubated ON with the corresponding secondary antibodies (1:50) conjugated to 20-nm gold particles (TED Pella Inc., Redding, CA, USA), contrasted with uranyl acetate and lead citrate, and observed through a Joel JEM-1011 transmission electron microscope.

### 2.10 dsRNA-based *Ehvps35* gene silencing

The knockdown (KD) of the *Ehvps35* gene was performed using the bacterial expressed double-stranded RNA (dsRNA) that was soaked with the trophozoites as described (Solis et al., 2009). Briefly, HT115 bacteria were transformed with *pL4440/Ehvps35* and grown at 37°C in LB broth in the presence of ampicillin (100 mg/ml) and tetracycline (10 mg/ml) (Takiff et al., 1989). The expression of *Ehvps35* dsRNA was induced by 1 mM IPTG overnight at 37°C. The dsRNA was then isolated from bacteria using a TRIzol reagent (Invitrogen) according to the manufacturer’s recommendations. DNase I (Invitrogen) and RNase A (Ambion, Austin, TX, USA) were added to remove the single-stranded RNA (ssRNA) and double-stranded DNA (dsDNA) molecules. The *Ehvps35* dsRNA was washed with isopropanol and ethanol, analyzed using agarose gel electrophoresis, and its concentration determined by spectrophotometry. Finally, the trophozoites (3 × 10^4^) in the TYI-S-33 medium were incubated with purified *Ehvps35* dsRNA molecules to a final concentration of 5 μg/ml and the cultures left for 48 h at 37°C, the time at which we previously determined was the silencing effect. Cells growing under standard conditions (without dsRNA) were used as the control.

### 2.11 Recycling of biotinylated surface proteins

For the recycling assays, we followed the protocol described by Ivaska et al. (2002) and Roberts et al. (2001), with some modifications. The trophozoites were incubated for 1 h at 37°C in serum-free TYI medium and then washed once with cold PBS. The cell surface proteins were labeled with 0.2 mg/ml of EZ-Link™ sulfo-NHS-SS-cleavable biotin (Thermo Scientific, Waltham, MA, USA) at 4°C for 30 min (Biller et al., 2014). After this time, the trophozoites were washed with cold PBS and incubated in TYI complete medium at 37°C for 15 min to allow internalization of the labeled proteins. To remove labeling from the cell surface proteins, the trophozoites were incubated in 100 mM dithiothreitol (DTT) for 30 min at 4°C, washed once with cold PBS, incubated again with TYI complete medium for 20 or 40 min at 37°C, and then observed through a laser confocal microscope.

For analysis of EhADH protein and Gal/GalNac lectin recycling, the protocol described above was followed, omitting the treatment with biotin and DTT and using an incubation step of 40 min at 37°C to follow the recycling of the proteins. Thereafter, the samples were processed to confocal microscopy using the antibodies and labeled secondary antibodies in order to follow the recycling of EhADH and Gal/GalNac. Moreover, for some experiments, the trophozoites were incubated with CHX (100 µg/ml) at 37°C for 30 min. Lastly, the samples were prepared for laser confocal microscopy and Western blot assays.

### 2.12 Three-dimensional model and molecular dynamics simulation of EhVps35

The three-dimensional (3D) model of EhVps35 was obtained on the I-TASSER server using the *Mus musculus* (PDBID:6VAB: B) and *H. sapiens* (PDB:7BLN:C) Vps35 as templates. The 3D model was refined through 200 ns of molecular dynamics stimulation (MDS) by NAMD2.8 (Phillips et al., 2020) with the force field CHARMM36 to create the topologies of the protein (Huang and Mackerell, 2013). The TIP3 model was applied for the water molecules. The system was solvated using the psfgen plug-in in the VMD program (Humphrey et al., 1996). Thereafter, 64,480 water molecules and 12 sodium ions were added to neutralize the system, which was minimized for 10,000 steps, followed by equilibration under constant temperature and pressure (NPT) conditions for 1 ns with the protein and lipid atoms restrained. Afterward, MDS was run for 200 ns, considering EhVps35 as a soluble protein, without position restraints under periodic boundary conditions (PBCs) and using an NPT ensemble at 310 K and 200 ns of MD simulation. The structures were visualized using the UCSF Chimera software. Once the best EhVps35 model was chosen, 3D structural alignment with the Vps35 from *H. sapiens* (PDB:7BLN:C) was developed to compare both proteins.

### 2.13 Protein–protein docking analysis

The published 3D predicted and refined structures (Montaño et al., 2017) of EhADH were used for docking experiments, whereas the 3D model of the EhVps35 protein was generated in this work. Snapshots were obtained using clustering analysis of the 200-ns MDS with the Carma software (Koukos and Glykos, 2013). Protein–protein docking was performed employing different conformers with the ClusPro server (Comeau et al., 2004; Kozakov et al., 2013). The conformers with the highest cluster members and the lowest energy were analyzed on the PDBSum server (Laskowski et al., 1997), while 3D model visualization was performed by the VMD program (Humphrey et al., 1996).

### 2.14 Immunoprecipitation assays

Immunoprecipitation was carried out using trophozoite lysates prepared in the presence of 10 mM Tris-HCl, 50 mM NaCl, and 100 mM protease inhibitors (i.e., PHMB, IA, NEM, and TLCK), followed by freeze–thawing cycles in liquid nitrogen and vortexing (Avalos-Padilla et al., 2015). In parallel, 200 μl of the recombinant protein G-agarose (rProtein-G; Invitrogen) was incubated with 100 μg of the mouse αEhVp35 antibodies or the pre-immune serum for 2 h at 4°C, with gentle stirring. Thereafter, the rProtein-G beads were washed with 0.5% bovine serum albumin (BSA) in PBS, followed by additional washes with PBS for 5 min, under gentle stirring, and centrifuged at 11,600 × *g* for 2 min. The trophozoite lysates (1 mg) were pre-cleared with 200 μl of rProtein-G (previously blocked with 2% BSA) and incubated for 2 h at 4°C under gentle stirring. The samples were then centrifuged at 11,600 × *g* to obtain the supernatant that was added to the rProtein-G previously incubated with antibodies. The preparations were incubated ON at 4°C and the beads recovered by centrifugation. After washes with PBS, 60 μl of 4× sample buffer (40% glycerol, 240 mM Tris-HCl, pH 6.8, 8% SDS, 0.04% bromophenol blue, and 5% β-mercaptoethanol) was added. The samples were boiled for 3 min and centrifuged again at 11,600 × *g* for 2 min at 4°C. The supernatant (30 μl) was loaded into 12% SDS-PAGE, subjected to Western blot assays, and revealed using the α-EhVps35, α-EhADH, α-Hgl Gal/GalNac, and α-actin antibodies.

### 2.15 Mass spectrometry analysis

Approximately 30 μg of the immunoprecipitated proteins was enzymatically digested according to the protocol reported by Ramírez-Flores et al. (2019). Afterward, the peptides were loaded into the Symmetry C18 Trap V/M precolumn (Waters, Milford, MA, USA), 180 μm × 20 mm, 100-Å pore size, and 5-μm particle size, and desalted using mobile phase A (0.1% formic acid in H_2_O) and mobile phase B (0.1% formic acid in acetonitrile) under the following isocratic gradient: 99.9% mobile phase A and 0.1% mobile phase B at a flow of 5 μl min^−1^ for 3 min. Subsequently, the peptides were loaded and separated on an HSS T3 C18 column (Waters), 75 μm × 150 mm, 100-Å pore size, and 1.8-μm particle size, using a UPLC ACQUITY M-Class (Waters) with the same mobile phases under the following gradient: 0 min with 7% mobile phase B, 121.49 min with 40% mobile phase B, 123–126.46 min with 85% mobile phase B, and 129–130 min of 7% mobile phase B, at a flow of 400 nl min^−1^ at 45°C. The spectra were acquired with a mass spectrometer using electrospray ionization and ion mobility separation with Synapt G2-Si (Waters) using a data-independent acquisition approach in HDMSE mode (Waters). The generated raw files containing the MS and MS/MS spectra were deconvoluted and compared using the ProteinLynx Global SERVER (PLGS) v3.0.3 software (Li et al., 2009) against a reversed *E. histolytica* database (downloaded from UniProt). All identifications had a reliability ≥95%.

### 2.16 Statistical analysis

The values for all experiments were expressed as the mean and standard error of at least three independent assays carried out in duplicate. Statistical analyses were performed with the GraphPad Prism v5.01 software using paired Student’s *t*-test (*p < 0.05, **p < 0.01, and ***p < 0.001).

### 2.17 Ethics statement

CINVESTAV fulfills the standard of the Mexican Official Norm (NOM-062-ZOO-1999), “Technical Specifications for the Care and Use of Laboratory Animals,” based on the Guide for the Care and Use of Laboratory Animals (“The Guide,” 2011, NRC, USA, with Federal Register no. BOO.02.03.02.01.908) awarded by the National Service for Agrifood Health, Safety and Quality (SENASICA). This organization verifies the state of compliance of such NOM in Mexico and is under the Ministry of Agriculture and Rural Development. The Institutional Committee for Animal Care and Use (IACUC/Ethics Committee) from CINVESTAV, the regulatory office for research protocol approval involving the use of laboratory animals, reviewed and approved all animal experiments (protocol no. 0505-12, CICUAL 001).

## 3 Results

### 3.1 *E. histolytica* presents a non-canonical retromer

**Figure 1:**
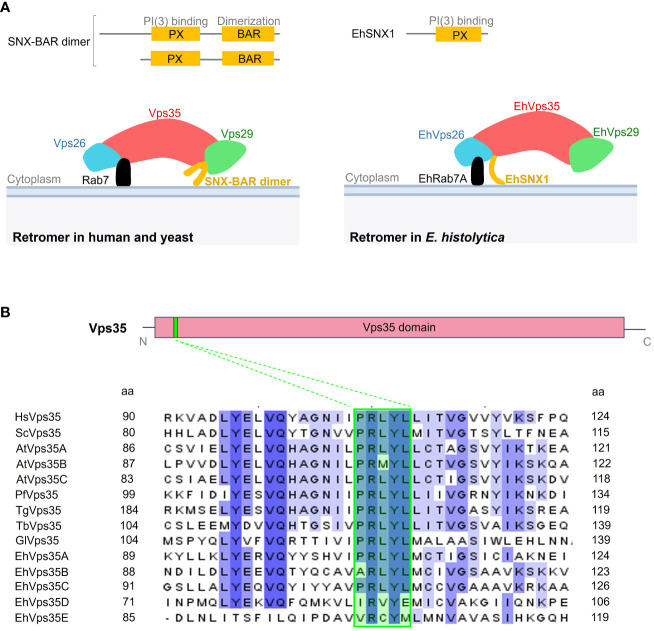
Comparative representation of the *Entamoeba histolytica* retromer with the human and yeast canonical structures. **(A)** *Upper section* shows the difference between the sorting nexin (SNX) proteins in *Homo sapiens*, *Saccharomyces cerevisiae*, and *E. histolytica*. *Below*: scheme illustrating the retromeric protein structure in these organisms. **(B)** Diagram of the Vps35 protein showing the Vps35 domain (*red box*) covering most of the protein and the PRLYL motif (*green box* in the sequences *below*) at the N-terminus. *Below*: amino acid sequence alignment of the fragment surrounding the PRLYL motif of Vps35 proteins from *H. sapiens* (*Hs*), *S. cerevisiae* (*Sc*), *Arabidopsis thaliana* (*At*), *Plasmodium falciparum* (*Pf*), *Toxoplasma gondii* (*Tg*), *Trypanosoma brucei* (*Tb*), *Giardia lamblia* (*Gl*), and *E. histolytica* (*Eh*). *Dashed lines* show the PRLYL motif marked in *green square* in all sequences. Shadow amino acids in these sequences indicate the identity and homology among the species according to intensity. The *numbers to the left* and *right* correspond to the regions of the alignment for each protein.

The canonical retromer in yeast and mammals is formed by Vps26, Vps29, Vps35, and SNX dimer (Vps5 and Vps17 in yeast and SNX1, SNX2, SNX5, and SNX6 in mammals). In contrast, only the EhVps26, EhVps29, EhVps35, and EhSNX1 proteins have been identified in *E. histolytica* (Nakada-Tsukui et al., 2005; Watanabe et al., 2020). In addition, unlike other SNX orthologs, EhSNX1 presents only the PX and not the BAR domain (**Figure 1A**). From what is known so far, EhVps26 is the only retromeric protein that binds to EhRab7A (**Figure 1A**) (Nakada-Tsukui et al., 2005), in contrast to the retromer of other species where Vps35 also interacts with Rab7 on the endosomal membranes (**Figure 1A**) (Seaman et al., 2009; Liu et al., 2012; Purushothaman et al., 2017).

In the *E. histolytica* genome, five EhVps35 isoforms (A–E) were found, but only two of them (EhVps35A and EhVps35C) were conserved in the complete PRLYL motif (**Figure 1B**), which allows the binding of Vps26 to Vps35 in yeast and mammalian cells. For this study, EhVps35C was selected as, so far, it is the only *E. histolytica* protein with experimental evidence of direct interaction with EhVps26 and EhVps29 (the other two proteins of the CSC complex) and it is translationally active (Marion et al., 2005; Nakada-Tsukui et al., 2005; Biller et al., 2014; Perdomo et al., 2015). However, the functionality of the other four EhVps35, found in AmoebaDB, cannot be discarded. In fact, there are reports on a functional Vps35 isoform without the complete PRLYL motif in *A. thaliana* (Jaillais et al., 2007; Hashiguchi et al., 2012; Zelazny et al., 2013).

### 3.2 EhVps35 is localized in the plasma membrane and in cytoplasmic vesicles

**Figure 2:**
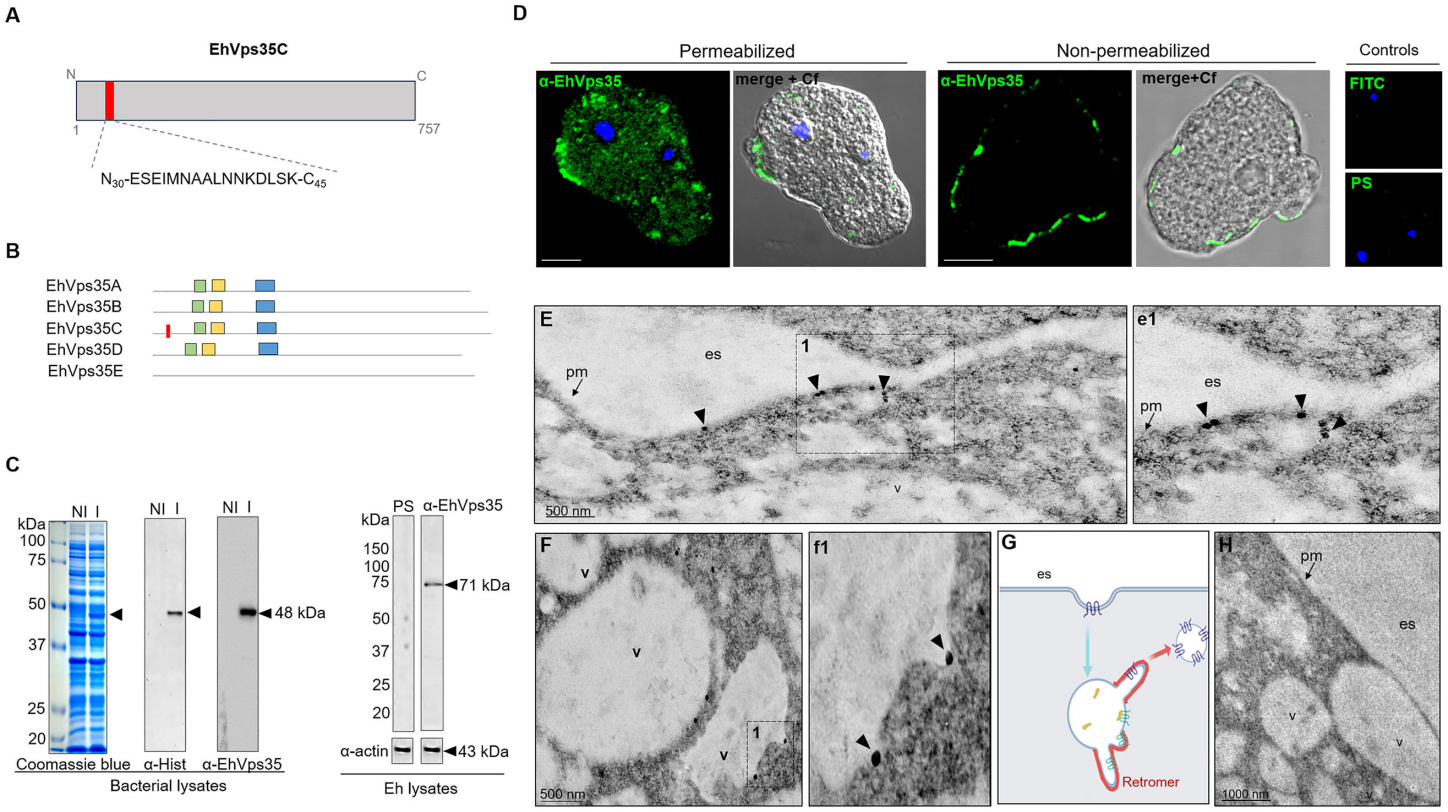
Cellular location of EhVps35. **(A)** Schematic representation of the amino acid sequence (*red box*) used to generate specific mouse α-EhVps35 antibodies. **(B)** Diagram of the conserved regions in EhVps35 isoforms, indicating in EhVps35C the region used to produce the α-EhVps35 antibodies. *Green box*, DLYELVQYYGNVIPRLYLMITVG; *yellow box*, LKDLIEMCRAVQHPTKGLFLRNYLL DCIK; *blue box*, YKTIILPRILEQVILCRDLVSQEYLMDAIIQAFPDEFHLKT; *red box*, peptide N_30_-ESEIMNAALNNKDLSK-C_45_. **(C)** Coomassie blue-stained gel (10% SDS-PAGE) containing the lysates of non-induced (*NI*) and induced (*I*) bacteria. *Arrows* denote the recombinant EhVps35 (*rEhVps35*). *Right*: Western blot assays of the NI and I transformed bacteria detected with the α-Hist and α-EhVps35 antibodies. Western blot assays of the lysates of trophozoites were developed with α-EhVps35 antibodies. *Arrow* denotes the EhVps35 protein. *PS*, pre-immune serum. Actin was used as the loading control. **(D)** Immunofluorescence of the EhVps35 protein using the α-EhVps35 antibodies and FITC-labeled α-mouse secondary antibodies in permeabilized and non-permeabilized trophozoites. DAPI (*blue*): nuclei. Controls: FITC secondary antibody and PS. *Scale bar*, 10 μm. **(E–F)** TEM localization of the EhVps35 protein in trophozoites. (*e1*) Magnification of the *discontinuous black square* in **(E)**, marked with *1*. **(F)** EhVps35 in vesicle membranes. (*f1*) Magnification of the *discontinuous black square* in **(F)** marked with *1*. **(G)** Scheme of the tubular structure formation that putatively will constitute the retromer. **(H)** Control trophozoites using only secondary antibodies. *es*, extracellular space; *pm*, plasma membrane; *v*, vesicles. *Arrowheads* denote EhVps35.

To study the cellular location and follow the movement of the EhVps35C (hereinafter EhVps35) protein in the trophozoites, we generated specific antibodies against an EhVps35 peptide (N_30_-ESEIMNAALNN KDLSK-C_45_) located at the amino terminal region of the protein and absent in other isoforms (**Figures 2A, B**). This polypeptide was commercially synthetized and injected into mice to generate antibodies (α-EhVps35). An EhVps35 fragment (1–476 amino acids) was also used to obtain the recombinant EhVps35 polypeptide (rEhVps35), labeled with a histidine tag (His-tag). The rEhVps35 was used to verify the specific recognition of the α-EhVps35 antibody in the Western blot assays. The α-Hist and α-EhVps35 antibodies recognized the same 48-kDa band in the lysates obtained from transformed bacteria (**Figure 2C**). Similarly, the α-EhVps35 antibody recognized a 71-kDa band in the trophozoite lysates (**Figure 2C**). The expected molecular weight of the EhVps35 protein, calculated from its amino acid sequence, was 87.2 kDa. However, the aberrant migration of EhVps35 was not surprising as this has been widely reported for the Vps35 protein in humans (Haft et al., 2000; Damen et al., 2006; Zhao et al., 2007; Fjorback et al., 2012) and for other EhVps in *E. histolytica* (Avalos-Padilla et al., 2015). The aberrant migration behavior could be explained by the protein structure and charge.

With a confocal microscope, in permeabilized trophozoites, the antibodies were shown to detect the EhVps35 protein in clusters, some of them located in vesicles scattered in the cytoplasm and near the plasma membrane (**Figure 2D**). Intriguingly, in non-permeabilized trophozoites, the EhVps35 protein appeared in the plasma membrane (**Figure 2D**), and TEM assays confirmed that it was located on the outer face of the plasma membrane and in vesicle membranes (**Figure 2E**, e1). Interestingly, EhVps35 was observed in elongated structures, suggesting that membrane remodeling possibly occurred at these points, an event necessary for protein recycling (**Figure 2F**, f1). The scheme in **Figure 2G** shows a diagram of the vesicle projections that could be forming the tubular retromer structures described in other systems (Bonifacino and Hurley, 2008; van Weering et al., 2010), and **Figure 2H** corresponds to the control treated with secondary antibody.

### 3.3 EhVps35 is involved in phagocytosis

Phagocytosis is one of the landmarks of trophozoite virulence. This function requires high activity in membrane remodeling to form the phagocytic cups and channels, the endosomes, the multivesicular bodies (MVBs), and other vesicles. At the same time, protein recycling is needed in the cell to reuse certain molecules and save energy. Thus, being a complex involved in the selection and recycling of proteins, the retromer may also be involved in phagocytosis.

We explored the fate of the EhVps35 protein under the RBC stimulus, tracking the protein with the α-EhVps35 antibodies and the FITC-labeled α-mouse secondary antibodies. Confocal images revealed that after 2 min contact of the trophozoites with RBCs, the EhVps35 protein was translocated at the contact site of both cells and was visualized decorating the adhered RBCs, as has been previously reported for the EhSNX1 and EhVps26 retromeric proteins (Nakada-Tsukui et al., 2005; Srivastava et al., 2017; Watanabe et al., 2020). EhVps35 was also found in the ingested RBCs, which confirms the high mobility of this protein from the vesicle membranes to the plasma membrane and *vice versa*, suggesting that the retromer acts in concert with other molecules during phagocytosis.

**Figure 3:**
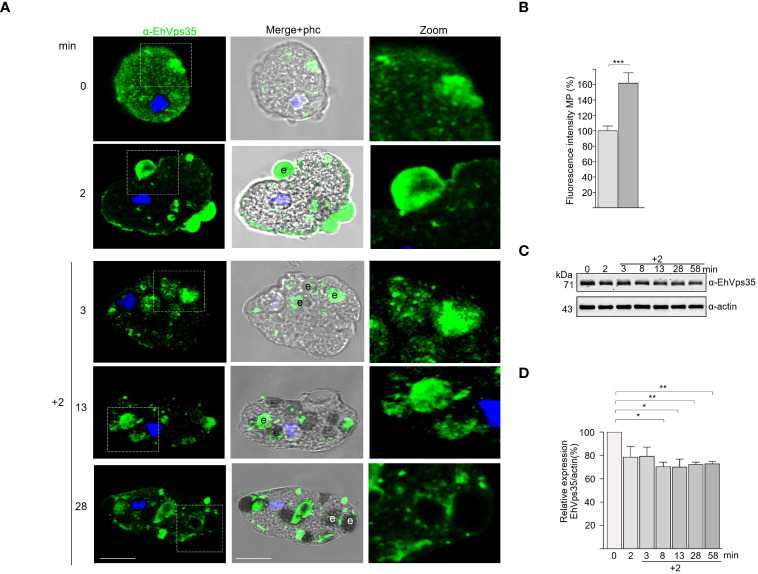
Fate of EhVps35 during phagocytosis. Trophozoites were incubated at 37°C with red blood cells (RBCs) (1:25) for 2 min, then non-ingested RBCs were removed by mild osmotic shock and incubated again in TYI medium as described in *Materials and methods*. **(A)** Immunofluorescent confocal images of the trophozoites at different times of phagocytosis using α-EhVps35 and the FITC-labeled secondary antibody. DAPI (*blue*): nuclei. *Left*: Incubation time in minutes. *Zoomed-in images* correspond to the regions marked with *white squares* in each row. *e*, erythrocytes. *Scale bar*, 10 μm. **(B)** Fluorescence intensity of the trophozoites in the plasma membrane at 0 and 2 min of phagocytosis. ***p < 0.001. **(C)** Western blot corresponding to the lysates of trophozoites at different times of phagocytosis. Actin was used as the loading control. **(D)** Densitometry analyses of the EhVps35 protein in Western blot assays at different times of phagocytosis. *p < 0.05; **p < 0.01.

In some experiments, after 2 min of phagocytosis, the adhered and non-ingested RBCs were removed by mild osmotic shock and then incubated again in TYI medium. These pulse-chase experiments were carried out to follow the fate of the already ingested cells, without the noise of other RBCs recently adhered and ingested. In these experiments, at 5 (2 + 3) and 15 (2 + 13) min, the antibodies again detected the EhVps35 protein in cytoplasmic vesicles, as well as on and in ingested erythrocytes. At 30 (2 + 28) min, the protein was located on and around the ingested erythrocytes and in phagosomes (**Figure 3A**). Our experiments showed that EhVps35, as are other retromeric proteins (**Figure 3B**) (Nakada-Tsukui et al., 2005, 2009; Watanabe et al., 2020), is located in the vesicle membranes that surround the ingested erythrocytes. Our results also evidenced that EhVps35, as are other proteins involved in phagocytosis (Avalos-Padilla et al., 2015; Galindo et al., 2021), follows the path of the ingested target cell.

Intriguingly, the Western blot assays of the lysates obtained from the trophozoite–RBC mixture revealed that the amount of EhVps35 protein apparently decreased during phagocytosis (**Figure 3C**). Densitometry analysis confirmed that, after 10 min of phagocytosis, the EhVps35 protein significantly decreased between 20% and 40% with respect to the 0 time (**Figure 3D**). This condition could be due to the partial degradation of the cellular amount of this protein during phagocytosis. Actin was used as a control to normalize the data for quantification (**Figures 3C, D**).

**Figure 4:**
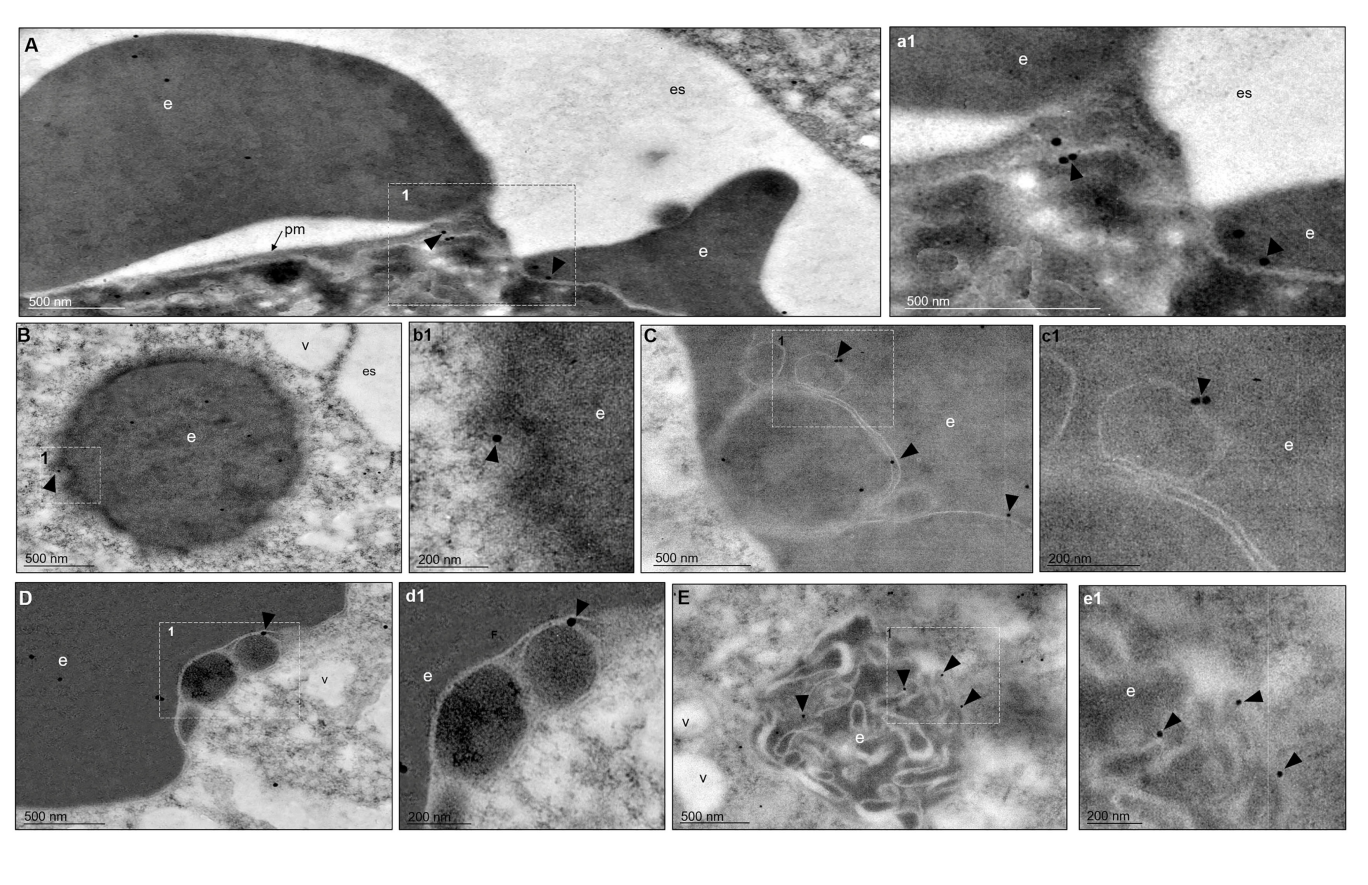
TEM localization of EhVps35 in trophozoites during erythrophagocytosis. **(A)** EhVps35 (*arrowheads*) in the plasma membrane and at the sites of contact of the trophozoites with RBCs. (*a1*) Magnification of the region marked with a *discontinuous white square* in **(A)**, marked with *1*. **(B)** Red blood cell (RBC)-containing phagosomes where the EhVps35 protein (*arrowheads*) was observed in the membrane. (*b1*) Magnification of the region marked with a *discontinuous white square* in **(B)**, marked with *1*. **(C, D)** EhVps35 protein (*arrowheads*) in white double-membrane structures inside phagosomes. (*c1*, *d1*) Magnification of the region marked with a *discontinuous white square* in **(C, D)**, marked with *1*. **(E)** Location of the EhVps35 protein (*arrowhead*) in white intricate structures (WIAs). (*e1*) Magnification of the region marked with a *discontinuous white square* in **(E)**, marked with *1*. *es*, extracellular space; *pm*, plasma membrane; *v*, vesicles; *e*, erythrocytes.

The TEM images, using gold-labeled secondary antibodies, confirmed that, after 5 min of phagocytosis, the EhVps35 protein was located at the contact sites of the trophozoites with RBCs, as observed in confocal images (**Figure 4A**, a1). Subsequently, EhVps35 was observed in the RBC-containing phagosomes. The gold label appeared mainly in the endosome and phagosome membranes, where it could have undergone an eventual membrane remodeling, which is necessary for protein recycling (**Figures 4B–D**, b1 - d1, respectively). In TEM assays, the RBC-containing phagosomes presented white intricate arrangements (WIAs) that have been reported in other studies on proteins involved in phagocytosis (Avalos-Padilla et al., 2015; Galindo et al., 2021). These WIAs could partially correspond to the digested hemoglobin that is inside these vesicular structures, which have not been thoroughly described. However, the location of the EhVps35 protein in the WIAs could also suggest that the retromer carried out its function of selecting proteins to recycle them to different cellular compartments (**Figure 4E**, e1). More experimental evidence is necessary to confirm this hypothesis.

### 3.4 EhVps35 participates in plasma membrane protein recycling

**Figure 5:**
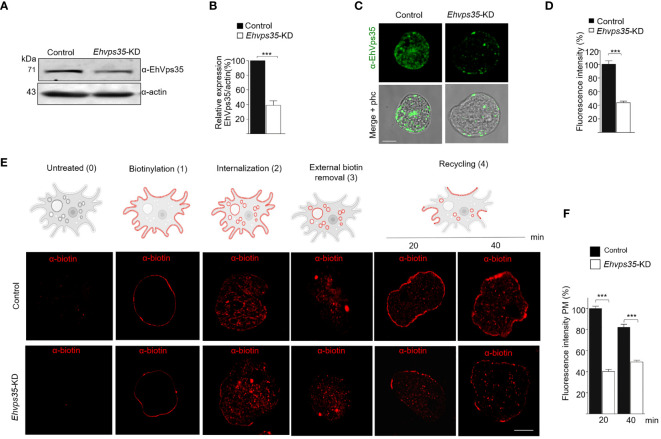
Recycling of the biotinylated proteins by EhVps35. **(A)** Western blot assays using non-silenced (control) and silenced (knocked down *Ehvps35*, *Ehvps35-KD*) trophozoites. **(B)** Densitometric analysis of the bands shown in **(A)** normalized against the actin protein bands. **(C)** Representative images of the confocal microscopy of the control and *Ehvps35*-KD trophozoites under basal condition using the α-EhVps35 and FITC-labeled antibodies (*green*). *Scale bar*, 10 μm. **(D)** Fluorescence intensity of the images in **(C)** measured in pixels. **(E)** The trophozoites were processed to follow the recycling process as described in *Materials and methods*. The *upper section* shows the scheme of the recycling process. *0*, Untreated cells. *1*, Biotin-labeled plasma membrane proteins (*red*) in the trophozoites at 4°C. *2*, Internalization of the labeled proteins at 37°C. *3*, Removal of the biotinylated proteins in the plasma membrane using DTT. *4*, Recycling to the plasma membrane of the stained proteins at 37°C. *Lower panel* shows confocal images of the control and *Ehvps35*-KD trophozoites following the steps in the upper scheme. *Scale bar*, 10 μm. **(F)** Fluorescence intensity measured in the plasma membrane of trophozoites at 20 and 40 min. ***p < 0.001.

To experimentally demonstrate that recycling occurred in the trophozoites in this study and that the EhVps35 protein is involved in this function, we knocked down the *Ehvps35* gene using dsRNA (Solis et al., 2009). Western blot assays using silenced trophozoites (*Ehvps35*-KD) evidenced that they expressed approximately 60% less EhVps35 than the control (**Figures 5A, B**). The results of the immunofluorescence assays corroborated these findings (**Figures 5C, D**). Subsequently, we used biotin (S-NHS-SS-Biotin) to label the plasma membrane proteins (Biller et al., 2014). Under a confocal microscope, the biotinylated trophozoites, maintained at 4°C, displayed the plasma membrane proteins uniformly labeled in both the control and *Ehvps35*-KD trophozoites (**Figure 5E**, 1). Thereafter, the trophozoites were incubated for 15 min at 37°C and the label internalized in both cell types (**Figure 5E**, 2). Interestingly, after 20 min incubation at 37°C, the labeled proteins returned to the plasma membrane in the control trophozoites, but not in the *Ehvps35*-KD trophozoites (**Figure 5E**, 4). The incubation time at 37°C was augmented to 40 min; however, recycling in the *Ehvps35*-KD trophozoites remained poor (**Figure 5E**, 4). After quantification of the fluorescence intensity in the plasma membrane, these assays confirmed that significant recycling reductions of 50% and 60% occurred in the *Ehvps35*-KD trophozoites compared to the control (**Figure 5F**). These results strongly suggest that the EhVps35 protein, even when located in the plasma membrane, does not participate in the introduction of the target cell, but has a vital role in protein recycling.

### 3.5 Three-dimensional model of EhVps35

**Figure 6:**
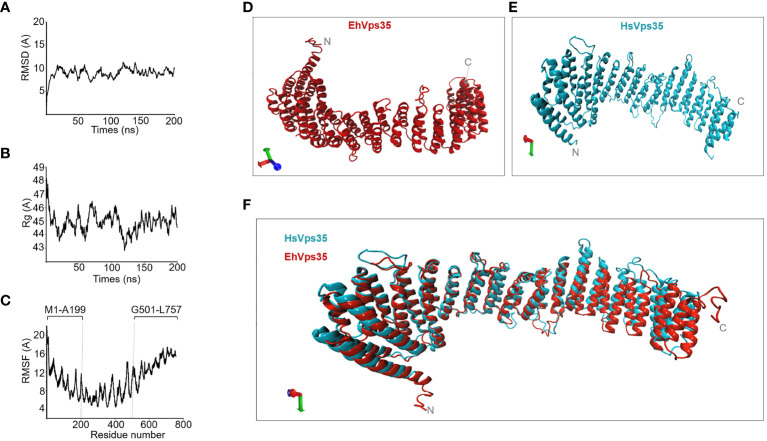
EhVps35 molecular dynamics stimulation (MDS) and the predicted 3D model. **(A)** Root mean square deviation (RMSD). **(B)** Radius of gyration (*R*_g_). **(C)** Root mean square fluctuation (RMSF). *Brackets* in RMSF indicate the most flexible regions. **(D)** Three-dimensional EhVps35 model. **(E)** Three-dimensional HsVps35 model. **(F)** Overlapping of EhVps35 and HsVps35.

To analyze the interaction of EhVps35 with the other membrane proteins involved in phagocytosis, the complete EhVps35 3D model was used. A previous report showed the 3D design of the EhVps35 carboxy terminus (Srivastava et al., 2017), but not the complete protein. Here, we obtained the full 3D protein structure from the I-TASSER server (**Figure 6D**). The structural alignment of this model exhibited a root mean square deviation (RMSD) of 1.07 Å with the HsVps35 (PDB:7BLN:C) crystal ortholog (**Figure 6F**). Analysis of the MDS trajectory measured using RMSD indicated that EhVps35 has an unstable trajectory oscillating ±3 Å, and it is independent of the simulation time (**Figure 6A**). The radius of gyration (*R*_g_) data confirmed that EhVps35 is an extended protein that is compacted during the MDS in the first 25 ns, after which is compacted again at 100 ns and expanded after 100–150 ns, remaining unchanged until the end of the simulation (**Figure 6B**). However, the root mean square fluctuation (RMSF) values showed a high movement of the protein in the N-terminus region from 1 to 199 amino acids and at the C-terminus from 501 to 757 amino acids (**Figure 6C**). This analysis showed that EhVps35 has the 3D model described for its orthologs as an α-solenoid protein (**Figure 6E**) (Hierro et al., 2007; Lucas et al., 2016) containing 33 α-helices (**Figure 6D**), confirming that EhVps35 is indeed an ortholog of the HsVps35 protein in humans.

### 3.6 *In silico* EhVps35 binding to EhADH

**Figure 7:**
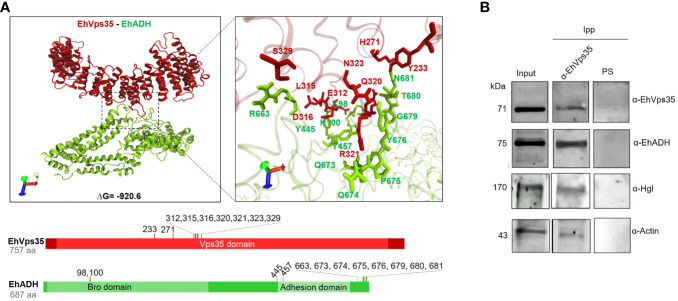
Molecular docking of EhVps35 and EhADH and immunoprecipitation assays. **(A)** Three dimensions of the conformational model of the binding of EhVps35 (*red*) and EhADH (*green*). *Lower panels*: Schematic representation of the amino acid residues that participate in the interaction between these molecules. **(B)** Immunoprecipitation of EhADH, Gal/GalNac, and actin using α-EhVps35. HM1 trophozoites in basal conditions were lysed and immunoprecipitated using the α-EhVps35 antibody or pre-immune serum (PS). Immunoprecipitated proteins were analyzed by Western blot using the α-EhVps35, α-EhADH, α-Hgl Gal/GalNac, and α-actin antibodies.

Once the EhVps35 model was obtained, docking analysis was carried out between EhVps35 and EhADH using the 3D EhADH model already reported (Montaño et al., 2017). The docking results predicted that EhVps35 interacts with EhADH with a global free energy of Δ*G* = −920.6, given by two salt bridges and 19 hydrogen bonds (**Figure 7A**). The EhVps35 residues that interacted with EhADH were H271, E312, L315, D316, Q320, R321, N323, S329, and Y233. On the other hand, in EhADH, these were K98, K100, Y445, Y457, R663, Q673, Q674, P675, Y676, G679, T680, and N681. The binding of EhADH to EhVps35 did not interfere with the Bro1 or adhesion domains in the EhADH protein as, except for the K98, K100, and Y457 amino acids that appeared located in these domains, most of the EhADH residues appeared situated at the C-terminus after the adhesion domain.

### 3.7 Experimental binding of EhVps35 to EhADH, Gal/GalNac lectin, and actin

Subsequently, we experimentally examined the EhVps35–EhADH binding with immunoprecipitation assays using the α-EhVps35 antibodies for precipitation and the total trophozoite extracts. The Western blot assays revealed that the α-EhADH antibodies recognized a 75-kDa protein in the immunoprecipitates, corresponding to the EhADH protein migration (**Figure 7B**). These results strongly suggest the association of EhVps35 with EhADH, a molecule that is actively mobilized during phagocytosis (Arroyo and Orozco, 1987; García-Rivera et al., 1999). Considering the docking analysis, the binding of EhVps35 and EhADH could occur by direct interaction; however, we cannot rule out that an indirect link can also happen.

In these experiments, we searched for the Gal/GalNac lectin, another membrane protein mobilized during virulence events (Petri et al., 2002; Frederick and Petri, 2005), and for actin as, during the retromer function, it binds to EhSNX-1 and EhSNX-1 interacts with the EhVps35 protein (Watanabe et al., 2020). In addition, Gal/GalNac lectin binds to actin and to other cytoskeletal proteins (McCoy and Mann, 2005). The EhVps35, EhADH, Gal/GalNac, and actin proteins appeared in the immunoprecipitates obtained with the α-EhVps35 antibody, strongly suggesting that they bind to each other. The pre-immune serum did not recognize any band (**Figure 7B**).

### 3.8 EhVps35 participates in the recycling of the EhADH and Gal/GalNac proteins

**Figure 8:**
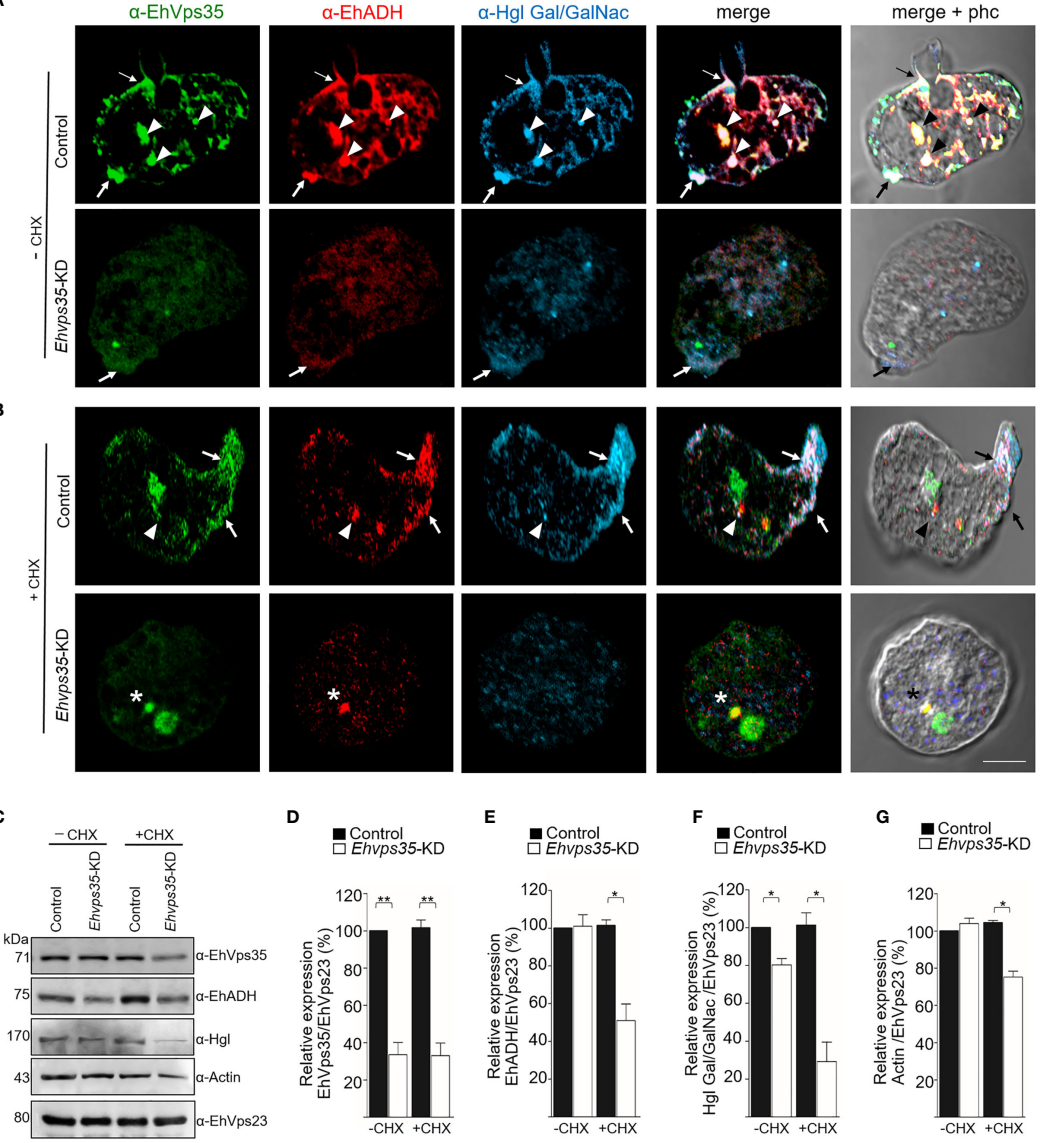
Recycling of Gal/GalNac and EhADH by EhVps35. **(A, B)** Confocal images of EhVps35 (*green*), EhADH (*red*), and Gal/GalNac (*blue*) during recycling as described in the *Material and methods*. Cycloheximide-treated (*+CHX*) and untreated (*−CHX*) trophozoites of the non-silenced (control) and silenced (knocked down *Ehvps35*, *Ehvps35-KD*) trophozoites. *Arrows* denote proteins that co-localized in the plasma membrane. *Arrowheads* indicate proteins that co-localized in the cytoplasm. *Asterisk* represents the co-localization of EhVps35 and EhADH in the cytoplasm of *Ehvps35*-KD trophozoites. *Scale bar*, 10 μm. **(C)** Western blot assays of the control and *Ehvps35*-KD trophozoites. *Right*: Name of the antibodies. *Left*: Molecular weights of the revealed protein. **(D–G)** Densitometric analysis of the bands shown in **(C)** normalized against the EhVps23 protein. *p < 0.05; **p < 0.01.

Once the association of EhVps35 with EhADH and Gal/GalNac was confirmed, we proceeded to investigate whether the retromer recycles these proteins using the *Ehvps35*-KD trophozoites and the respective antibodies against the two proteins. Firstly, under a confocal microscope, the α-EhVps35, α-EhADH, and α-Hgl Gal/GalNac antibodies in the control trophozoites were detected to co-localize in the plasma membrane and in some cytoplasmic regions (**Figure 8A**), suggesting the formation of EhADH and Gal/GalNac protein complexes associated with EhVps35. In contrast, in the *Ehvps35*-KD trophozoites, the fluorescent signals of the three antibodies were poor (**Figure 8A**). Weak labeling was also observed in dots scattered in the cytoplasm, without visible labels in the plasma membrane. The three antibodies also exhibited very poor co-localization in *Ehvps35*-KD trophozoites (**Figure 8**). These experiments demonstrated that, when the EhVps35 protein is poorly expressed, the recycling of EhADH and Gal/GalNac lectin is affected, strongly supporting the participation of EhVps35 in this event.

Subsequently, to avoid interference of the proteins that were *de novo* synthesized and then transported to the plasma membrane, we used CHX to stop the protein synthesis and followed only those proteins present in the cell at the beginning of the recycling experiments. In the confocal images of control cells, the α-EhVps35, α-Hgl Gal/GalNac, and α-EhADH antibodies were again detected in the plasma membrane (**Figure 8B**, arrow) and in some dots in the cytoplasm (**Figure 8B**, arrowhead). The three proteins appeared to mainly co-localize in clusters in the plasma membrane, suggesting that CHX did not affect their interaction, nor the morphology of the trophozoites, as can be observed in the phase-contrast images (**Figures 8A, B**). Cell viability was neither affected, which was measured using trypan blue exclusion. In contrast, the *Ehvps35*-KD cells showed poor fluorescence labels, and their cellular location was also disturbed due to the proteins remaining scattered in the cytoplasm, but not in the plasma membrane (**Figure 8B**). Several *Ehvps35*-KD trophozoites presented a large cytoplasmic vesicle labeled by antibodies against the remnant EhVps35 and EhADH, and protein co-localization appeared poor and in small cytoplasmic vesicles (**Figure 8B**). These results confirmed that the recycling of EhADH and Gal/GalNac proteins was affected by the knockdown of the *Ehvps35* gene.

To further explore the decrease of the EhADH and Gal/GalNac proteins observed in *Ehvps35* knockdown, Western blot assays were performed using the lysates from untreated and CHX-treated trophozoites. In untreated *Ehvps35*-KD trophozoites, the Gal/GalNac protein significantly decreased (**Figures 8C, D, F**), but the EhADH and actin proteins did not show apparent changes (**Figures 8C, E, G**). In contrast, in CHX-treated *Ehvps35*-KD trophozoites, the EhADH, Gal/GalNac, and actin proteins significantly decreased between 20% and 60% with respect to the control trophozoites (**Figures 8C–G**). The α-EhVps23 antibody was used to normalize the data. These results suggest a possible degradation of EhADH, Gal/GalNac, and actin in the absence of the EhVps35 protein and the treatment with CHX.

### 3.9 In *Ehvps35-KD* trophozoites, the EhADH protein is localized mainly in acidic vesicles

**Figure 9:**
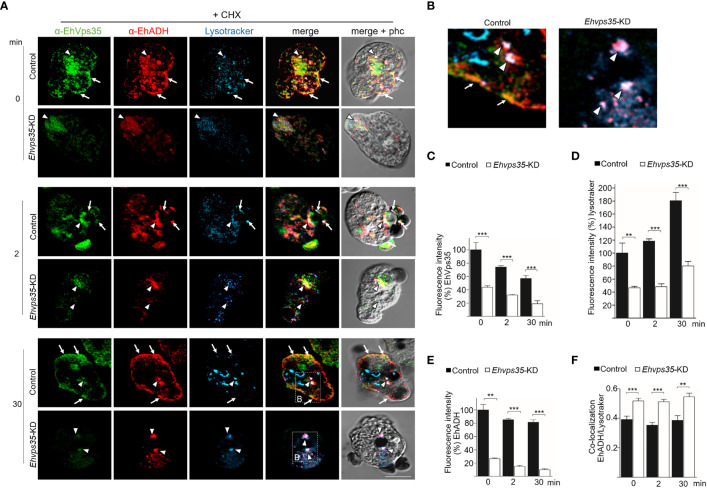
Recycling and co-localization of EhADH in acidic structures. **(A)** Localization of the EhVps35 protein (*green*), EhADH protein (*red*), and the acidic structures (*blue*) in cycloheximide-treated (*+CHX*) non-silenced (control) and *Ehvps35*-KD trophozoites. *Arrows* indicate the co-localization sites of the EhVps35 and EhADH proteins in the plasma membrane. *Arrowheads* denote the co-localization sites of the EhVps35 and EhADH proteins with acidic structures in the cytoplasm. *Scale bar*, 10 μm. **(B)** Magnification of the regions marked with a *discontinuous white square* in **(A)** at 30 min. **(C–E)** Fluorescence intensity of EhVps35, EhADH, and LysoTracker measured in pixels. **(F)** Pearson’s coefficient for EhADH and LysoTracker co-localization. **p < 0.01; ***p < 0.001.

Previous studies have shown that EhADH can be located in acidic vesicles during phagocytosis (Castellanos-Castro et al., 2016), suggesting that a portion of this protein is conducted to degradation and another portion is recycled, as shown here (**Figures 8A, B**). Thus, LysoTracker was used to investigate whether EhADH appeared in acidic vesicles in the control and the CHX-treated *Ehvps35*-KD trophozoites. In basal conditions, the control trophozoites showed that the EhVps35 and EhADH proteins co-localized, but their co-localization with LysoTracker was low. Interestingly, the CHX-treated *Ehvps35*-KD trophozoites showed higher co-localization of both proteins with LysoTracker (**Figures 9A–F**). These results suggest that a greater degradation of both proteins could have occurred in *Ehvps35*-KD trophozoites compared to the control. After phagocytosis (2 min), in the control trophozoites, the EhVps35 and EhADH proteins moved to the contact site with RBCs. In contrast, the *Ehvps35*-KD trophozoites appeared poorly labeled and the protein co-localization with LysoTracker was also poor (**Figures 9A–F**). In the control cells, after 30 min of phagocytosis, the EhVps35 and EhADH proteins re-localized to the plasma membrane and co-localized with LysoTracker in some RBC-containing phagosomes, suggesting that, during phagocytosis, a portion of them were conducted to degradation. In contrast, the CHX-treated *Ehvps35*-KD trophozoites presented fewer acidic vesicles, the EhADH protein did not return to the plasma membrane, and EhVps35 and EhADH remained in small points in the cytoplasm, with some of them co-localizing with LysoTracker (**Figures 9A, F**). Quantitative data of the fluorescence intensity of the EhVps35 and EhADH proteins and the acidic vesicle marker showed a significant reduction (60%–80%) in *Ehvps35*-KD trophozoites compared to the control cells (**Figures 9B–D**). These results strengthened the assumption that EhADH is degraded in the absence of the EhVps35 protein, and these remark the importance of the retromer in phagocytosis, recycling and preserving the proteins participating in these events.

### 3.10 Actin disruption in *Ehvps35*-KD trophozoites

**Figure 10:**
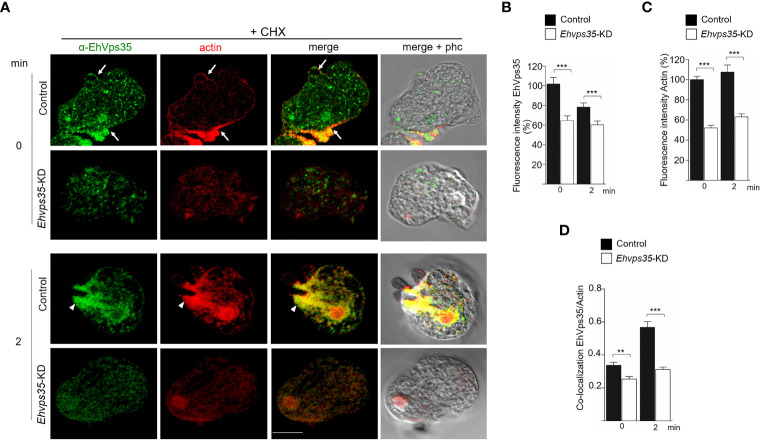
Actin cytoskeleton disruption during the phagocytosis in *Ehvps35* knockdown (*Ehvps35*-KD) trophozoites. **(A)** Localization of EhVps35 (*green*) and actin (*red*) in the non-silenced (control) and silenced (*Ehvps35*-KD) trophozoites after 0 and 2 min of phagocytosis. *Arrows* denote the co-localization of the EhVps35 and actin proteins near the plasma membrane. *Arrowheads* indicate the co-localization of EhVps35 and actin in phagocytic cups and the channels. *Scale bar*, 10 μm. **(B, C)** Fluorescence intensity of EhVps35 and actin measured in pixels. **(D)** Pearson’s coefficient for EhVps35 and actin co-localization. **p < 0.01; ***p < 0.001.

The Western blot assays revealed a decrease of actin in *Ehvps35*-KD trophozoites (**Figures 8C, D**). To explore whether the actin cytoskeleton was affected by the *Ehvps35* gene silencing, confocal microscopy assays were performed using CHX-treated trophozoites. In basal conditions, in the control trophozoites, EhVps35 and F-actin again co-localized mainly near the plasma membrane and in cytoplasmic structures, as described (Manich et al., 2018a). In contrast, *Ehvps35*-KD trophozoites presented the diminished fluorescence intensity of F-actin and disorganization of the actin cytoskeleton (**Figures 10A–C**). After 2 min of phagocytosis, the control trophozoites displayed the EhVps35 and F-actin proteins relocated to the contact site with the RBCs, decorating the phagocytic cups and channels (**Figure 10A**). However, these structures were not observed in the *Ehvps35*-KD trophozoites, suggesting that the silencing of the *Ehvps35* gene affected the structuring of the actin cytoskeleton, which is necessary in the formation of phagocytic cups and channels (**Figures 10A, B**). Quantitative data of the fluorescence intensity of the EhVps35 and F-actin proteins showed a reduction of 40%–70% in *Ehvps35*-KD trophozoites compared to the control cells (**Figures 10B, C**). Co-localization analysis between EhVps35 and F-actin showed an increase in the control trophozoites after 2 min of phagocytosis, while the *Ehvps35*-KD trophozoites remained constant. Furthermore, in *EhVps35*-KD trophozoites, the co-localization between EhVps35 and F-actin was significantly lower compared to that in the control trophozoites (**Figure 10D**). These results suggest that CHX treatment did not affect the structure of the actin cytoskeleton. To obtain more insights on the relationship between the EhVps35 protein and actin cytoskeleton structuring, the immunoprecipitates with α-EhVps35 antibodies were analyzed using mass spectrometry. EhVps35-bound proteins were selected after comparison to previous proteomic analyses of the actin cytoskeleton in *E. histolytica* (Manich et al., 2018; Rath and Gourinath, 2020). The results revealed 33 cytoskeleton-associated proteins, including actin and cytoskeleton modulator proteins (**Table 1**).

**Table 1:** Proteins associated with EhVps35 and related to the actin cytoskeleton.

Protein	Accession number
Actin	EHI_140120/EHI_126190/EHI_161200/EHI_182900
Profilin 1	EHI_176140
CAP	EHI_136150/EHI_081430
ARPC2	EHI_199690
ARP2	EHI_111050
ARPC1	EHI_045000
ARP3	EHI_198930
Myosin-II	EHI_110180
Bin1/Endophilin	EHI_187770
Cortexillin	EHI_083140
Cortexillin	EHI_104560
Coronin 1/CRN12a	EHI_083590
Coronin 2/CRN12b	EHI_105330
α-actinin1	EHI_146140/EHI_164430
Filopodin	EHI_080740
LimD	EHI_194520
EhLIM-A	EHI_096420
LimC	EHI_110280
Actin-binding Rho-activating protein	EHI_187110
F-actin bunding C-domain	EHI_189930/EHI_103860/EHI_004550/EHI_086690/EHI_010570
TolA	EHI_159620
TolA-like	EHI_052780
Rab2C	EHI_067850
RabC3	EHI_143650
Rab1A	EHI_108610
RabX11	EHI_177520
RabC1	EHI_153690
Rab GDP dissociation inhibitor	EHI_167060
Rho GTPase	EHI_129750
Ras GTPase	EHI_058090
α-Tubulin	EHI_010530

Trophozoite proteins were immunoprecipitated with α-EhVps35 antibodies. The EhVps35-bound proteins were identified by mass spectrometry. Comparative analysis with previous proteomic analyses of the actin cytoskeleton was performed (Manich et al., 2018; Rath and Gourinath, 2020).

**Figure 11:**
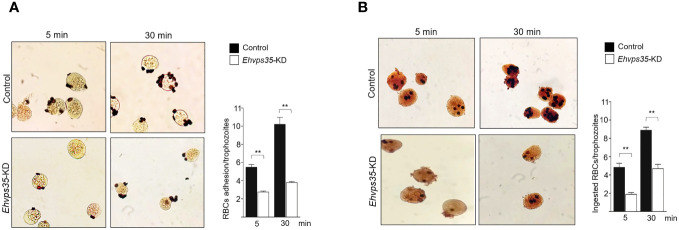
Reduction in the phagocytosis rate of *Ehvps35* knockdown (*Ehvps35*-KD) trophozoites. Novikoff-stained trophozoites after 5 and 30 min of incubation with red blood cells (RBCs) in non-silenced (control) and silenced (*Ehvps35*-KD) trophozoites. **(A, B)** RBC adherence (4°C) and phagocytosis (37°C), respectively. On the *right of each image*, the mean of the number of attached and ingested RBCs by trophozoites counted in 100 randomly chosen trophozoites in three independent experiments is shown. **p < 0.01.

Finally, to examine the importance of the EhVps35 in phagocytosis, for its role in surface protein recycling and the actin cytoskeleton structure required for this event, the adhesion efficiency and the rate of phagocytosis were evaluated using the silenced trophozoites. The results showed a decrease of 50%–63% in the adhesion efficiency and 53%–61% in the rate of phagocytosis in *Ehvps35*-KD trophozoites at 5 and 30 min, respectively, with respect to the results of the control trophozoites, which were taken as 100% (**Figures 11A, B**). These results strengthened the assumption that the EhVps35 protein is involved in the phagocytosis of trophozoites, probably due to its active participation in protein recycling and in the structuring of the actin cytoskeleton.

## 4 Discussion

The retromer has been widely studied in humans due to its involvement in neurodegenerative diseases (Zhang et al., 2018). However, in parasitic protozoan, further research has yet to be conducted to determine whether this protein complex participates in virulence events and, therefore, whether it is a target to defeat parasites. In *T. gondii*, the retromer is crucial for the biogenesis of secretory organelles and to maintain the morphology of parasites; thus, several authors agree in the fact that the retromer indeed participates in *T. gondii* virulence (Sangaré et al., 2016; Gras et al., 2019). In the case of *E. histolytica*, there are reports suggesting that the retromer is involved in phagocytosis, an important virulence mechanism in this parasite (Nakada-Tsukui et al., 2005; Srivastava et al., 2017; Watanabe et al., 2020). The retromer in these protozoa differs from the canonical one, described in yeast and mammals. The main differences are that, in both protozoa, the SNX-BAR is absent, which suggests that the membrane remodeling mechanisms in these parasites could differ from those of the classical pathway (**Figure 1A**) (Nakada-Tsukui et al., 2005; Koumandou et al., 2011; Sangaré et al., 2016; Watanabe et al., 2020). More knowledge is needed on the characteristics and function of each of the elements that construct the protozoan retromer to reveal their participation in cellular functions, particularly in virulence. This knowledge will also help in understanding the evolutionary process that this conserved complex has gone through.

In this paper, we have studied the structure and function of the EhVps35 protein, the core of the CSC complex, as it binds EhVps29 and EhVps26, maintaining the complex and allowing the retromer function. Our work is relevant to the advance of the knowledge of the *E. histolytica* retromer as:

i. It presented data on the EhVps35 3D model;
ii. It provided evidence on the interaction of the retromer with the EhADH and Gal/GalNac lectin and actin proteins;
iii. It provided experimental evidence that the *E. histolytica* retromer carries out surface protein recycling and that EhVps35 is necessary to carry out this function;
iv. It showed that EhVps35 recycles two highly involved proteins in the host–parasite relationship: the EhADH adhesin (Arroyo and Orozco, 1987; García-Rivera et al., 1999; Bañuelos et al., 2012) and the Gal/GalNac lectin (Frederick and Petri, 2005);
v. It provided data on the influence of EhVps35 on the structuring of the actin cytoskeleton during phagocytosis, providing further experimental data on phagocytosis being carried out by a concatenated chain of events in which several molecules participate, including EhADH, an accessory protein of the ESCRT machinery; and
vi. The data presented here strongly support the retromeric proteins also participating in phagocytosis. The knockdown of the *Ehvps35* gene revealed that, when one of the elements of phagocytosis is altered, the whole event is disrupted.

The main function of the retromer is the protein recycling from the endosomes to the *trans*-Golgi apparatus and the plasma membrane after selection of the internalized proteins. Thus, although its presence in the plasma membrane was not surprising, as it occurs with other retromeric proteins in mammals (Kerr et al., 2005), the presence of EhVps35 in the outer plasma membrane is intriguing because there is no information on its function in this location. More experimental data are needed to investigate whether the retromer acts together with other molecules from the first contact of the trophozoite with the target cell.

The knockdown of the *Ehvps35* gene (*Ehvps35*-KD) in trophozoites presented interesting results on the role of this protein, not only in protein recycling but also during the process of phagocytosis. The adhesion efficiency and the rate of phagocytosis were reduced in *Ehvps35*-KD trophozoites, possibly due to the absence of the EhVps35 protein, which caused a decrease in the EhADH and Gal/GalNac recycling. However, we cannot discard gene expression coordination of the chain links involved in phagocytosis. This is an assumption based on the work of Ocádiz-Ruiz et al. (2016), where it was found that silencing of the EhADH gene affects the expression of EhCP112 and *vice versa*. Moreover, EhADH is a scaffold protein; therefore, impairment in its localization could affect the recruitment of other proteins necessary for phagocytosis (Arroyo and Orozco, 1987; García-Rivera et al., 1999; Frederick and Petri, 2005; Bañuelos et al., 2012; Avalos-Padilla et al., 2015; Galindo et al., 2021). In addition, our results showed that EhVps35 co-localized with EhADH and F-actin in the early and late stages of phagocytosis. As a consequence, *Ehvps35*-KD trophozoites showed less ability to form phagocytic cups and channels. This event may be related to the decrease in actin exhibited by the CHX-treated trophozoites. This could be due to: i) the ligand attachment to the Gal/GalNac lectin in the plasma membrane, triggering a series of events including cytoskeletal reorganization and signal transduction, hence producing, in *Ehvps35*-KD trophozoites with less Gal/GalNac lectin, an actin disruption (Marion et al., 2004; McCoy and Mann, 2005; Meza et al., 2006; Emmanuel et al., 2015); ii) the CHX treatment in these trophozoites could quickly activate signaling mediated by proteins of the Rho family, which regulates the structuring and degradation of actin (Guild et al., 2002; Darvishi and Woldemichael, 2016); or iii) the poor interaction between EhVps35 and EhSNX1, which could have caused the EhSNX1protein to not bind to the initiator of actin polymerization (AP2/3) (Watanabe et al., 2020), strongly suggesting that the *E. histolytica* retromer is essential for the organization of the actin cytoskeleton. In fact, this has been suggested for other organisms (Harbour et al., 2010; McGough et al., 2014; Zavodszky et al., 2014). Furthermore, 33 actin cytoskeletal proteins directly or indirectly bound to EhVps35 were identified by mass spectrometry (**Table 1**).

The decrease of EhVps35 caused poor acidification of the endosomes (**Figure 9**), which could lead to a decreased rate of phagocytosis. In *S*. *cerevisiae*, *Drosophila melanogaster*, and *Dictyostelium discoideum*, the retromer is vital for the lysosomal pathway as this complex recycles the V-ATPase subunit, a complex responsible for regulating cell acidification (Carnell et al., 2011; Finnigan et al., 2011; Maruzs et al., 2015; Ye et al., 2020). Gilmartin et al. (2017) demonstrated that, when ATPase activity was inhibited, the rate of phagocytosis in *E. histolytica* decreased. Furthermore, it has also been reported that EhVps26 and EhVps29 mutant trophozoites showed a decrease in CP activity, proteins that require acidic structures to be functional (Nakada-Tsukui et al., 2005; Srivastava et al., 2017).

**Figure 12:**
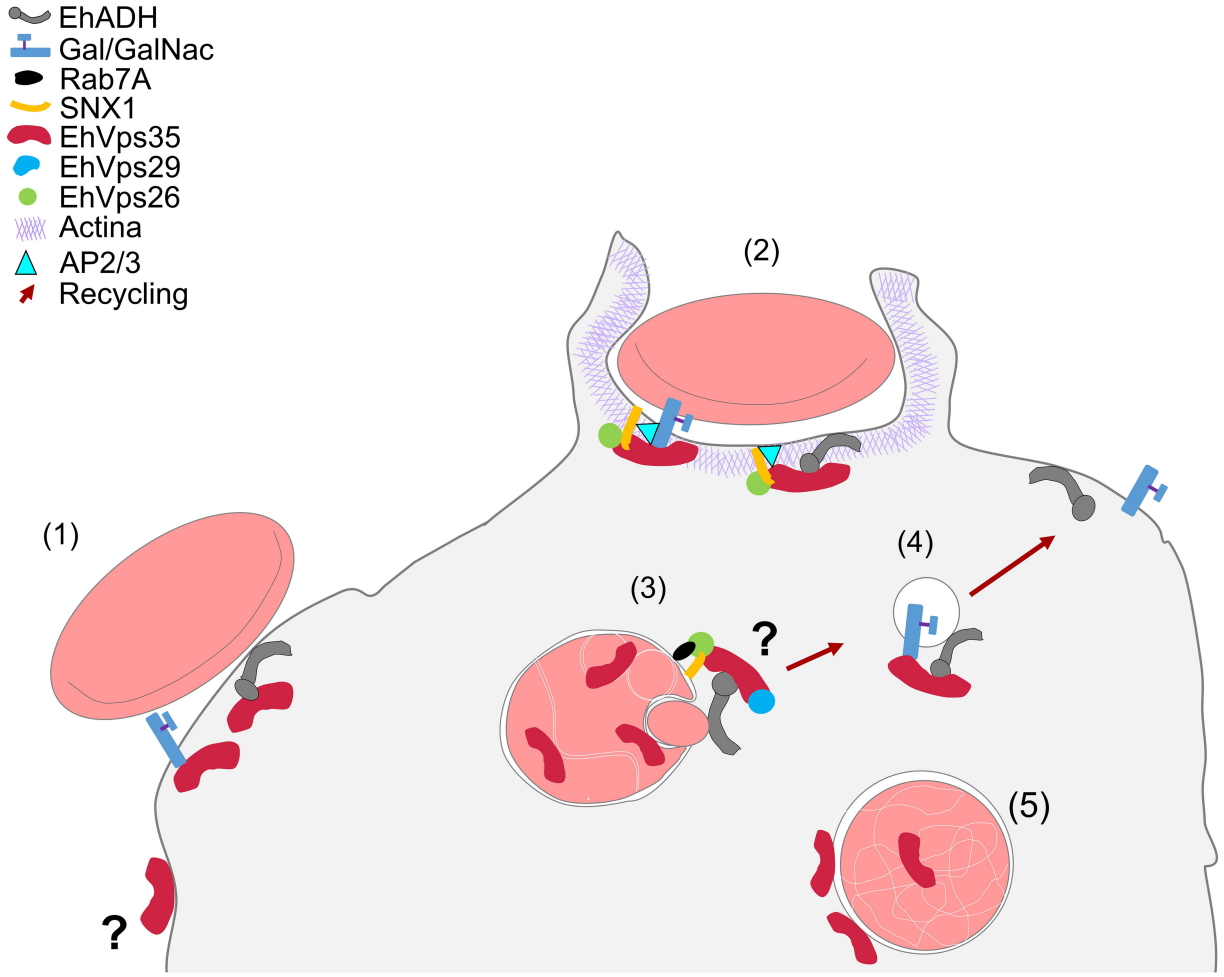
Working model of the involvement of EhVps35 in EhADH and Gal/GalNac recycling in phagocytosis and in actin cytoskeleton structuring. (*1*) Adhesion of RBCs to trophozoites produced cellular signaling for phagocytic cup formation with the recruitment of several proteins, including EhVps35, EhADH, Gal/GalNac, and SNX1. (*2*) Phagocytic cup formation in the presence of the initiator of actin polymerization (AP2/3) during interaction with SNX1, EhVps26, and EhVps35. (*3*) EhVps35 in phagosomes in white intricate arrangements (WIA) in the phagosomes, probably selecting the proteins that will be recycled. (*4*) Recycling of the vesicle toward the plasma membrane, which carries the EhADH and Gal/GalNac proteins. (*5*) EhVps35 in lysosomes.

In conclusion, our work provides an overview on the complex interactions between EhVps35, EhADH, Gal/GalNac, and actin during protein transport to different compartments in the phagocytosis of *E. histolytica* (**Figure 12**). This primitive protozoan is an excellent model for studying vesicular transport to further explore the retromer functions in phagocytosis and other virulence mechanisms. In addition, the roles of EhVps35 in protein recycling and in *E. histolytica* phagocytosis will reveal new targets for anti-amoebiasis drug design.

## Data availability statement

The datasets presented in this study can be found in online repositories. The names of the repository/repositories and accession number(s) can be found below: https://www.uniprot.org/, Q96QK1; https://www.uniprot.org/, P34110; https://www.uniprot.org/, C4M8Z4; https://www.uniprot.org/, C4M4G0; https://www.uniprot.org/, A0A8U0WP17; https://www.uniprot.org/, C4LWG4; https://www.uniprot.org/, C4M3E8; https://www.uniprot.org/, Q7X659; https://www.uniprot.org/, F4I0P8; https://www.uniprot.org/, A8R7K9; https://www.uniprot.org/, Q8IIQ6; https://www.uniprot.org/, S7USD8; https://www.uniprot.org/, Q38C17; https://www.uniprot.org/, Q6Y0Y2.

## Ethics statement

The human samples used in this study were acquired from primarily isolated as part of your previous study for which ethical approval was obtained. Written informed consent for participation was not required from the participants or the participants’ legal guardians/next of kin in accordance with the national legislation and institutional requirements.

## Author contributions

JD: Conceptualization, Writing – original draft, Investigation, Methodology. RJ: Supervision, Writing – original draft, Methodology. SM: Writing – original draft, Methodology, Software. DT: Writing – original draft, Methodology. EO: Conceptualization, Funding acquisition, Supervision, Writing – original draft, Writing – review & editing.
